# Serum microRNAs in ASD: Association With Monocyte Cytokine Profiles and Mitochondrial Respiration

**DOI:** 10.3389/fpsyt.2019.00614

**Published:** 2019-09-10

**Authors:** Harumi Jyonouchi, Lee Geng, Gokce A. Toruner, Shannon Rose, Sirish C. Bennuri, Richard E. Frye

**Affiliations:** ^1^Department of Pediatrics, Saint Peter’s University Hospital (SPUH), New Brunswick, NJ, United States; ^2^Clinical Cytogenetics, Department of Hematopathology, MD Anderson Cancer Center, Houston, TX, United States; ^3^Department of Pediatrics, Arkansas Children’s Hospital Research Institute, Little Rock, AR, United States; ^4^Department of Pediatrics, Phoenix Children’s Hospital, Phoenix, AZ, United States

**Keywords:** autism spectrum disorder, cytokine, microRNA, mitochondria, monocytes, serum

## Abstract

Our previous research has shown that purified peripheral blood monocytes (PRMo) from individuals who are diagnosed with autism spectrum disorders (ASDs) and have innate immune abnormalities reveal altered interleukin-1ß (IL-1ß)/IL-10 ratios. We also found, in separate studies, that microRNA (miRNA) expression in PBMo and mitochondrial respiration in peripheral blood mononuclear cells (PBMCs) differed in the IL-1ß/IL-10-based ASD subgroups. This study explored whether serum miRNAs are associated with both altered innate immune responses and changes in mitochondrial respiration as a link of regulatory mechanisms for these two common abnormalities in ASD subjects. Serum miRNA levels were examined by high-throughput deep sequencing in ASD and non-ASD control sera with concurrent measurement of PBMo cytokine production and mitochondrial respiration by PBMCs. ASD samples were examined as a whole group and with respect to the previously defined IL-1ß/IL-10-based ASD subgroups (high, normal, and low groups). Serum miRNA levels differed between the overall ASD sera (N = 116) and non-ASD control sera (N = 35) and also differed across the IL-1ß/IL-10-based ASD subgroups. Specifically, miRNA levels were increased and decreased in eight and nine miRNAs, respectively, in the high-ratio ASD subgroup (N = 48). In contrast, the low- (N = 25) and normal- (N = 43) ratio ASD subgroups only showed decreased miRNAs levels (18 and 10 miRNAs, respectively). Gene targets of the altered miRNAs in the high and/or low IL-1β/IL-10 ratio ASD subgroups were enriched in pathways critical for monocyte functions and metabolic regulation. Gene targets of the altered miRNAs in all the ASD subgroups were enriched in pathways of neuronal development and synaptic plasticity, along with cell proliferation/differentiation. ASD subgroup-specific associations were observed between serum miRNA expression and IL-1ß/IL-10 ratios, mitochondrial respiration, and monocyte cytokine profiles (IL-10, CCL2, and TNF-α). In summary, our results indicate that serum levels of select miRNAs may serve as promising biomarkers for screening and monitoring changes in innate immunity and mitochondrial respiration in ASD.

## Background

Repeated genetic studies suggest that only about 10–20% of individuals with autism spectrum disorder (ASD) have a well-defined gene mutation (1). The etiology of ASD, in the great majority of patients, is believed to be due to complex interactions of genetic and environmental factors during critical periods of brain development. Given the diverse effects of such genetic and environmental factors, it is not surprising that disease outcome and therapeutic options are highly heterogeneous in ASD population. However, there are few objective biomarker(s) useful for assessing clinical outcomes, responses to intervention measures, and/or the underlying effects of genetic/environmental factors in ASD. Many individuals with ASD do not respond well to the first-line behavioral and pharmacological interventions. The identification of objective biomarkers will be especially important for such poor responders.

ASD has a high incidence of co-morbid conditions that affect multiple organs other than the brain. Such co-morbid conditions are often associated with immune dysfunction and/or inflammation (2). In fact, there is mounting evidence that chronic inflammation plays a role in the pathogenesis of ASD (2–4). Individuals with ASD and immune-mediated inflammation affecting multiple organs may be referred to as having “inflammatory autism.”

Innate immune abnormalities are one of the most frequently reported immune problems in children with ASD (5–7). Our previous research has indicated that a subgroup of children with ASD show evidence of chronic inflammation associated with innate immune abnormalities which are best reflected in changes in IL-1ß/IL-10 ratios produced by ASD PBMo (8). We have also reported that IL-1ß/IL-10 ratios can be used to categorize children with ASD into three subgroups: those with high, normal, and low IL-1ß/IL-10 ratios, as compared to controls. We then further showed that monocyte cytokine profiles, miRNA expression by PBMo, and mitochondrial respiration by PBMCs, and behavioral symptoms differed across the IL-1ß/IL-10-based ASD subgroups (8–10). Our findings indicate that IL-1ß/IL-10 ratios along with PBMo miRNA expression may serve as a biomarker for screening alteration in innate immune responses in ASD. However, these parameters are not practical for screening measures, since the measurement of IL-1ß/IL-10 ratios by PBMo and miRNA expression are time-consuming, require a large amount of blood, and fresh blood samples must be processed rapidly. In addition, these types of analysis are not readily available in most clinical settings. We thus turned to serum biomarkers. Serum miRNAs typically require much smaller amounts of blood and serum samples that can be stored for prolonged time periods before processing. Thus, miRNA serum biomarkers, if applicable, would be ideal in screening for alteration of innate immune responses, with functional assays acting as second-line measures in pre-screened ASD subjects.

It has been revealed that serum miRNAs can mediate innate immune responses. Exosomal miRNAs secreted by monocyte/macrophage lineage cells are stable in body fluid and affect functions in cells that take up exosomal miRNAs (11–13). Since exosomal miRNAs cross the blood–brain barrier, they can affect brain cells. The major cellular sources of serum miRNAs are platelets and monocyte-macrophage lineage cells (13). In fact, serum miRNA levels are candidate biomarkers for disease status in various neuro-inflammatory diseases, including Alzheimer’s disease, Parkinson’s disease, and multiple sclerosis (11, 14–17).

Considering the above described findings, we hypothesized that serum miRNA levels are altered in the IL-1ß/IL-10-based ASD subgroups and are associated with altered monocyte cytokine profiles and mitochondrial respiration in individuals with ASD. To test our hypotheses, we examine the association between serum miRNA levels, PBMo cytokine profiles, and mitochondrial respiration in PBMCs in individuals with ASD. Typically developing (TD), non-ASD subjects were served as controls. The results obtained indicate that serum levels of certain miRNAs may serve as easily accessible biomarkers for screening innate immune abnormalities in ASD.

## Materials and Methods

### Study Subjects

The study followed the protocols approved by the Institutional Review Board, Saint Peter’s University Hospital, New Brunswick, NJ, United States. In this study, both ASD and non-ASD, TD subjects were enrolled, and the signed consent forms were obtained prior to entering the study. Consent was obtained from parents if participant was a minor (<18 years old) or parents had custody. For ASD children, we also assessed whether they had history of food allergy (FA), asthma, allergic rhinitis (AR), specific antibody deficiency (SAD), or seizure disorders. Subjects diagnosed with chromosomal abnormalities, other genetic diseases, or well characterized chronic medical conditions involving a major organ, were excluded from the study. Subjects with common minor medical conditions such as AR, mild to moderate asthma, and eczema were not excluded from the study. The some study subjects are overlapped with previously published manuscript (10), but there is no overlap of the data presented.

#### ASD Subjects

ASD subjects (N = 105) were recruited from the Pediatric Allergy/Immunology Clinic. Diagnosis of ASD was made at various autism diagnostic centers, including ours. The ASD diagnosis was based on the Autism Diagnostic Observation Scale (ADOS) and/or Autism Diagnostic Interview-Revisited (ADI-R), and other standard measures. ASD subjects were also evaluated for their behavioral symptoms and sleep habits with the Aberrant Behavior Checklist (ABC) (18) and the Children’s Sleep Habits Questionnaires (CSHQ) (19), respectively. Information regarding cognitive ability and adaptive skills were obtained from previous school evaluation records performed within 1 year of enrollment to the study, using standard measures such as Woodcock-Johnson III test (for cognitive ability) and Vineland Adaptive Behavior Scale (VABS) (for adaptive skills) (20).

#### Non-ASD Controls

TD, non-ASD control subjects (N = 34) were recruited from the pediatric Allergy/Immunology and General Pediatrics Clinics. These subjects were not reported to have any medical conditions included in the exclusion criteria and self-reported not to have seizure disorders or known immunodeficiency.

Demographic information of the study subjects is summarized in **Table 1**. There were no differences between females and males by two tailed Mann–Whitney test with regard to mitochondrial respiration parameters, and monocyte cytokine profiles examined in both ASD and non-ASD groups, as reported before (10).

**Table 1:** Demographics of ASD subjects and non-ASD controls.

	ASD^1^ subjects (N = 105)	Non-ASD controls (N = 35)
Age (years)^2^ Median, range Average ± SD	10.6 (2.2−21.5) 11.3 ± 5.4	13.3 (3.9−29.7) 15.5 ± 7.8
Gender (M:F)	88:17 (83.8%:16.2%)	27:8 (77.1%:22.9%)
Ethnicity	AA 7, Asian 23, mixed 1, W 74	Asian 3, mixed 4, W 28
Cognitive activity <1%	78/105 (74.3%)	0
Disturbed sleep	36/105 (34.3%)	0
GI symptoms	73/105 (69.5%)	0
History of NFA	64/105 (61.0%)	0
Seizure disorders	13/105 (12.4%)	0
Specific antibody deficiency	20/105 (19.0%)	0
Allergic rhinitis	21/105 (20.0%)	Unknown
Asthma	14/105 (13.3%)	Unknown

AA, African American; ASD, autism spectrum disorder; F, female; GI, gastrointestinal; M, male; NFA, non-IgE-mediated food allergy; SD, standard deviation; W, Caucasian.

^2^Age at the time of study enrollment was shown.

#### Diagnosis of FA

IgE-mediated FA was diagnosed with reactions to offending food, by affecting the skin, GI, and/or respiratory tract immediately (within 2 h) after intake with positive prick skin testing (PST) reactivity, and/or presence of food allergen–specific serum IgE. Non-IgE-mediated FA (NFA) was diagnosed if GI symptoms resolved, following implementation of a restricted diet (i.e., avoidance of offending food), and symptoms recurred with re-exposure to offending food (21). NFA patients are per definition, non-reactive to PST, and negative for food allergen–specific, serum IgE (21).

#### Diagnosis of Asthma and AR

AR and allergic conjunctivitis (AC) were diagnosed with positive PST reactivity, and/or presence of allergen-specific IgE in the serum, accompanied by clinical features consistent with AR and AC (22, 23). Asthma diagnosis was based on the guidelines from the Expert Panel Report 3 (24).

#### Antibody Deficiency Syndrome

SAD was diagnosed by the absence of protective levels of antibody (Ab) titers (>1.3 µg/ ml) to more than 11 of 14 serotypes of *Streptococcus pneumonia*, following a booster dose of Pneumovax^®^ or PCV13, a standard diagnostic measure for SAD (25).

### Sample Collection

Blood samples were obtained by venipuncture. For the non-ASD control subjects, only one sample was obtained. For select ASD subjects (N = 10), samples were obtained at two time points to assess variability of serum miRNA levels. Venipuncture was conducted by the physician. The site of venipuncture was numbed by applying a topical lidocaine/prilocaine cream (EMLA cream^®^), if requested by parents.

### Cell Cultures

PBMCs were isolated by Ficoll-Hypaque density gradient centrifugation. PBMo were purified by negatively selecting PBMo depleting T, B, natural killer, and dendritic cells from PBMCs, using magnetic beads labeled with anti-CD3, CD7, CD16, CD19, CD56, CD123, and glycophorin A (monocyte separation kit II—human, Miltenyi Biotec, Cambridge, MA, United States).

PBMo cytokine production was assessed by incubating purified PBMo (2.5x10^5^ cells/ml) overnight using a panel of agonist of toll-like receptors (TLRs) to reflect effects of microbial byproducts commonly encountered in real life. Lipopolysaccharide (LPS), a TLR4 agonist, is a representative endotoxin, reflecting a common pathway of innate immune responses by gram negative [G (−)] bacteria. Zymosan, a TLR2/6 agonist, is a representative innate immune stimulus from G (+) bacteria and fungi. CL097, a TLR7/8 agonist, mimics stimuli from ssRNA viruses, common respiratory pathogens causing respiratory infection, such as influenza. These stimuli have been widely used for testing innate immune responses. PBMos were incubated overnight with LPS (0.1 µg/ml, GIBCO-BRL, Gaithersburg, MD, USA), zymosan (50 µg/ml, Sigma-Aldrich, St. Luis, Mo), and L097 (water-soluble derivative of imidazoquinoline, 20 µM, InvivoGen, San Diego, CA, USA), in RPMI 1640 with additives as previously described (26). Overnight incubation (16–20h) was adequate to induce the optimal responses in this setting. The culture supernatant was used for cytokine assays.

Levels of pro-inflammatory (tumor necrosis factor-α [TNF- α], IL-1β, IL-6, and IL-12p40) and counter-regulatory [IL-10, transforming growth factor-ß (TGF-ß) and soluble TNF receptor II (sTNFRII)] cytokines were measured by enzyme-linked immuno-sorbent assay (ELISA); 10–100 µl/well supernatants were used for ELISA. The OptEIA^™^ Reagent Sets (BD Biosciences, San Jose, CA, USA) were used for ELISA of IL-1ß, IL-6, IL-10, IL-12p40, and TNF-α. For sTNFRII, and TGF-ß ELISA, reagents were obtained from BD Biosciences and R & D (Minneapolis, MN, USA). IL-23 ELISA Kit was purchased from eBiosciences, San Diego, CA. Intra- and inter-variations of cytokine levels were less than 5%.

### Categorizing ASD Samples on the Basis of IL-1β/IL-10 Ratios

Previously, we reported that changes in the IL-1ß/IL-10 ratios best reflect altered cytokine profiles and miRNA expression by PBMo (8, 9). We divided ASD samples into subgroups based on the IL-1ß/IL-10 ratios produced by ASD PBMo, following the criteria used in our previous study as outlined below (9).

#### High IL-1β/IL-10 Ratio

IL-1ß/IL-10 ratios > +2 standard deviation (SD) than control cells under at least one culture condition and/or > +1SD under more than two culture conditions.

#### Normal IL-1β/IL-10 Ratio

IL-1ß/IL-10 ratios between −1SD < IL-1ß/IL-10 ratios < +1SD under all the culture conditions, or +1SD < IL-1ß/IL-10 ratios < +2SD under only one culture condition.

#### Low IL-1β/IL-10 Ratios

IL-1ß/IL-10 ratios < −1SD under at least one culture condition.

As for 10 ASD subjects whose samples were taken at two time points, most subjects were categorized in the same group with two time-point analyses. One subject revealed a low ratio at one time point and a normal ratio at one time point; this subject was categorized in the low-ratio group.

### Assays of Mitochondrial Function

PBMCs (2 × 10^6^ cells) were suspended in biofreezing medium (90% heat-inactivated fetal calf serum and 10% DMSO) and kept in −20°C for about 1 h and then transferred to −80°C degree freezer and kept until shipment. Samples were sent to Dr. R. Frye’s laboratory on dry ice where the Seahorse Extracellular Flux (XF) 96 Analyzer (Seahorse Bioscience, Inc., North Billerica, MA, USA) was used to measure oxygen consumption ratio (OCR; pMol/min), which is an indicator of mitochondrial respiration, in real time in live PBMCs. ATP-linked respiration (ALR), proton leak respiration (PLR), maximal respiratory capacity (MRC), and reserve capacity (RC) were obtained by sequentially adding pharmacological agents and measuring the changes in OCR (27, 28). Measures of mitochondrial respiration were derived by the sequential addition of pharmacological agents to the respiring cells. For each parameter, three repeated OCR measurements were made over an 18-min period. First, baseline cellular OCR is measured, from which basal respiration is derived by subtracting non-mitochondrial respiration. Next oligomycin, an inhibitor of complex V, is added, and the resulting OCR is used to derive ALR (by subtracting the oligomycin rate from baseline OCR) and PLR by subtracting the resulting OCR from non-mitochondrial respiration. Next carbonyl cyanide-p-trifluoromethoxyphenyl-hydrazon (FCCP), a protonophore, is added to collapse the inner membrane gradient, driving the eletron transport chain (ETC) to function to its maximal rate, and MRC is derived by subtracting non-mitochondrial respiration from the FCCP OCR. Lastly, antimycin A, a complex III inhibitor, and rotenone, a complex I inhibitor, are added to shut down ETC function, revealing the non-mitochondrial respiration. RC is calculated by subtracting basal respiration from MRC.

Both ALR and MRC are measures of the ability of ETC to produce ATP, the molecule that carries energy to other areas of the cell to support vital functions. The ETC is not absolutely efficient. In fact, the ETC is a major source of the production of reactive oxygen species (ROS), which can damage the mitochondria and the cell if produced in excess. In order to reduce the production of ROS, the ETC can “leak” some of its energy. This “leak” is measured by PLR and makes the ETC less efficient at producing energy. In general, PLR should increase as more ATP is produced since the production of ATP does create ROS. The ratio of the above described measures of ATP production, specifically ALR and MRC to PLR, can provide a measure of the efficiency of the ETC. Theoretically, this ratio would be very high with very efficient mitochondrial function and very low in dysfunctional mitochondria where a great amount of ROS is produced to make energy.

### Serum miRNA Sequencing

Small RNA libraries were prepared by using the Small RNA Library Prep Kit for Illumina (Norgen Biotek Corp., Thorold, ON, Canada) according to the manufacturer’s instructions. A 6% Novex^®^ TBE PAGE Gel (Life Technologies, Carlsbad, CA) was used to separate the indexing PCR product and cut the specific library band size after staining with SYBR^®^ Gold Nucleic Acid Gel Stain (Life Technologies). The gel piece was crushed by centrifugation at 14,000 × g for 2 min in a Gel Breaker Tube (IST Engineering, Milpitas, CA). Libraries were quantified by the High Sensitivity DNA Analysis Kits on the Agilent 2100 Bioanalyzer System (Agilent Technologies, Santa Clara, CA). Libraries (4 nM) were sequenced on the Illumina NextSeq 550 at Norgen Biotek Corp. CA, using Illumina’s NextSeq 500/550 High Output v2 Kit (75 cycles). Sequencing data were converted to the FASTQ format and then used for read mapping to the hg38 human genome version on the Genboree Workbench’s exceRpt small RNA-seq pipeline (v4.6.2) (29). Read quality was assessed after adapter trimming by FASTQC to filter out poor quality score reads (lower than 30 on the PHRED scale). The UniVec and human rRNA sequences are excluded from the reads before mapping to miRNAs using miRBase version 21. Raw read counts were further analyzed using R (v3.4.0). Multiple R packages were used in the analysis including EdgeR (v3.18.1) for filtration based on a count per million (CPM) corresponding to a minimum raw count of 5. Read per million was used as a normalization method where the read of a specific small miRNA was divided by the total numbers of mapped reads and multiplied by one million. For statistical analysis in several groups and assessment of correlation, the trimmed mean of M-values (TMM) normalization method was used as TMM normalized read counts (CPM) (30). Differential expression (DE) between two groups was used to predict the relative miRNA expression variations between two groups. TMM normalized counts were used to achieve accurate DE analysis with reduced false-positive rate (FDR) with the use of EdgeR statistical software package for DE analysis (31). The Benjamini–Hochberg procedure was used adjust the FDR.

### Statistical Analysis

For comparison of two sets of numerical data, a two tailed Mann–Whitney test was used. For comparison of several sets of numerical data, Kruskal–Wallis test was used. For differences in frequency between two groups, the Fisher exact test was used. For differences in frequency among multiple groups, the chi-square test and the likelihood ratio were used. A linear association between two data sets was determined by Spearman test. P value of less than 0.05 was considered nominally significant. Co-variance analysis was done with the use of general linear model for a fixed factor or for a variable factor. NCSS 12 (NCSS, LLC. Kaysville, UT) was used for statistical analysis.

For determining the gene targets of specific miRNAs, microRNA Data Integration Portal (mirDIP) was used (http://ophid.utoronto.ca/mirDIP/index.jsp) (32). Putative gene targets with an integrated score of 0.3 and higher were further analyzed using Database for Annotation, Visualization, and Integrated Discovery (DAVID) (https://david.ncifcrf.gov/home.jsp) (32). Functional annotation analysis was performed to see enrichment for genes belonging to specific KEGG (33) pathways and UniProt and Gene Ontology keywords. Only the categories scored p < 0.05 after Benjamini–Hochberg Multiple hypothesis correction were considered significant in the initial analysis and further analyzed by Bonferroni test.

## Results

### Serum miRNA Levels in ASD and Non-ASD Control Subjects

High-throughput deep sequencing of serum miRNA in ASD and non-ASD samples revealed over two-fold differences of expression (DE) in several miRNAs between ASD and non-ASD control serum samples (4 up-regulated and 14 down-regulated). We then analyzed levels of 27 miRNAs in the ASD sera in comparison with non-ASD controls when expressed as TMM normalized readouts (CPM) (**Table 2**). Eighteen miRNAs are those that revealed significant DEs in the whole ASD samples as compared to non-ASD controls. Additional nine miRNAs are those that revealed significant DEs in one or two ASD subgroups as compared to non-ASD controls with readouts (>7.0).

**Table 2:** miRNA TMM normalized read counts in ASD and non-ASD control sera.

miRNA^1^	ASD subjects	Non-ASD controls	Mann–Whitney test
**All ASD subjects**			
miR-206	332.7 ± 296.1^2^	34.5 ± 73.8	0.004
miR-576-3p	90.3 ± 86.8	605.9 ± 1,257.3	0.0138
miR-193a-5p	151.0 ± 179.1	817.0 ± 1,325.9	0.00025
miR-27a-5p	17.3 ± 23.8	66.0 ± 101.4	0.0031
miR-379-5p	77.2 ± 70.8	223.9 ± 260.5	0.001
miR-134-5p	147.9 ± 150.3	397.4 ± 486.7	0.00013
miR-184	32.5 ± 90.3	12.4 ± 27.5	0.001
miR-574.-3p	10.5 ± 8.2	26.3 ± 74.5	0.215
miR-382-5p	190.0 ± 154.2	484.1 ± 428.6	0.0001
miR-223-5p	93.6 ± 87.3	25.6 ± 74.5	0.215
miR-7-5p	140.4 ± 171.1	312.9 ± 345.7	0.0107
miR-103a-3p	624.8 ± 319.0	1,342.6 ± 1,342.1	0.0309
miR-378a-3p	1,633.9 ± 1,666.2	3,491.2 ± 3,865.8	0.0103
miR-3614-5p	36.6 ± 59.5	75.3 ± 103.6	0.0977
miR-873-3p	29.3 ± 71.2	60.2 ± 120.1	0.9332
miR-4732-5p	130.8 ± 118.3	64.8 ± 72.6	0.00018
miR 193b-5p	41.4 ± 76.0	20.8 ± 28.2	0.0019
miR-433-3p	17.5 ± 14.6	35.1 ± 44.9	0.25
**ASD high IL-1ß/IL-10 ratio group**			
miR-423-5p	53,761.7 ± 60,519	27,956.6 ± 26,599.0	0.0013
miR-483-5p	161.7 ± 254.3	84.2 ± 180.9	0.0337
miR-320b	610.8 ± 631.1	368.0 ± 249.9	0.0021
miR-20a-5p^3^	291.6 ± 525.6	468.9 ± 580.3	0.6736
miR-320d	156.5 ± 222.4	103.1 ± 106.9	0.027
**ASD normal IL-1ß/IL-10 ratio group**			
miR-370-3p	173.1 ± 243.1	267.3 ± 350.4	0.0887
**ASD low IL-1ß/IL-10 ratio group**			
miR-100-5p	10,289.1 ± 6,580.2	18,560.4 ± 3,324,941	0.8507
miR-99b-5p	8,976.9 ± 6,580.2	15,433.6 ± 5,556.1	0.0548
miR-93-5p	171.6 ± 156.1	281.6 ± 278.1	0.1037

^1^TMM normalized values of miRNAs differentially expressed (DE) in all the ASD subjects and ASD subgroups, as compared to non-ASD controls were shown. In select nine ASD subjects, serum samples were obtained at two time points as shown in **Figure 1** and, in one ASD subject, serum samples were obtained at three time points.

^2^Expressed as TMM normalized read counts (CPM) ± standard deviation (SD).

^3^miR-20a-5p was also differentially expressed in the low-ratio ASD subgroup.

**Figure 1:**
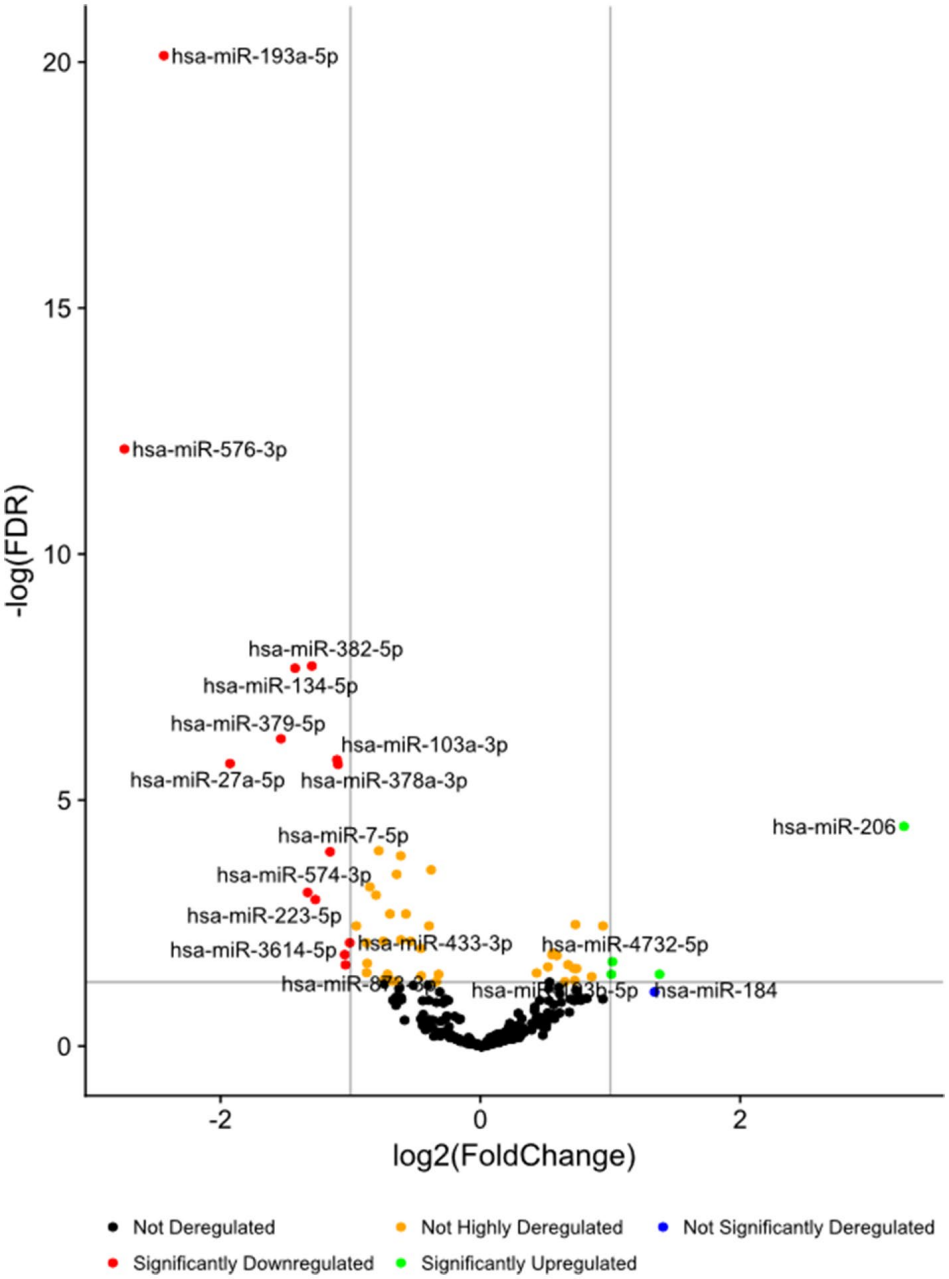
Volcano plots of differential expression of serum miRNAs in all the ASD samples combined, in comparison with non-ASD controls.

In these 27 miRNAs, we determined whether clinical features including gender, age, ASD severity, and medication use, affect serum miRNA levels. Serum miRNAs of two, four, two, two, and three miRNAs differed according to gender, and use of neuroleptics, ADHD medications, AEDs, and SSRIs, respectively. These results were shown in **Supplemental Table 1**. Only two miRNAs (miR-193b-5p and miR-320b) revealed significant associations between miRNA readouts and ages (p < 0.05) in both ASD subjects and non-ASD subjects. The clinical parameters did not affect differences between ASD subjects and non-ASD controls by co-variance analysis (p < 0.05), except for miR-206 which revealed the effects of the use of AEDs; ASD subjects (N-30) revealed lower miR-206 levels as compared to ASD subjects without AEDs (**Supplemental Table 1**).We also determined if there are any differences on the basis of ASD severity. In 7/27 miRNAs, there were nominally significant differences in serum miRNA levels with ASD severity (**Table 3**). Four out of seven miRNAs differed in levels in the whole ASD subjects as compared to non-ASD controls. Co-variance analysis rejected the effects of ASD severity on differences in these four miRNA serum levels between ASD and non-ASD subjects (p < 0.05).

**Table 3:** Serum miRNA levels on the basis of ASD severity.

miRNA	Severe (N = 68)	Moderate (N = 24)	Mild (N = 24)	Kruskal–Wallis
miR-27a-5p^2^	20.1 ± 24.7^1^	9.9 ± 10.5	17.0 ± 29.4	0.0349
miR-382-5p^2^	178.2 ± 160.8	242.4 ± 165.3	204.8 ± 114.2	0.0353
miR-223-5p^2^	118.6 ± 95.3	64.0 ± 74.7	80.7 ± 65.5	0.0043
miR-103a-3p^2^	685.5 ± 365.0	528.6 ± 247.7	549.0 ± 181.3	0.0328
miR-20a-5p^3^	358.9 ± 670.9	202.8 ± 174.5	189.5 ± 85.5	0.0275
miR-370-3p^3^	141.2 ± 202	237.8 ± 362.7	198.9 ± 191.6	0.0185
miR-93-5p^3^	193.3 ± 174.8	132.3 ± 114.5	149.5 ± 127.3	0.0155

^1^Serum miRNA levels were expressed as TMM normalized read counts (CPM) ± SD as detailed in the methods section. In nine and one ASD subjects, serum samples were obtained at two and three time points respectively.

^2^These serum miRNAs were differently expressed in ASD subjects as compared to non-ASD controls.

^3^These serum miRNAs were differently expressed in the ASD subgroups as compared to non-ASD controls.

**Figure 2:**
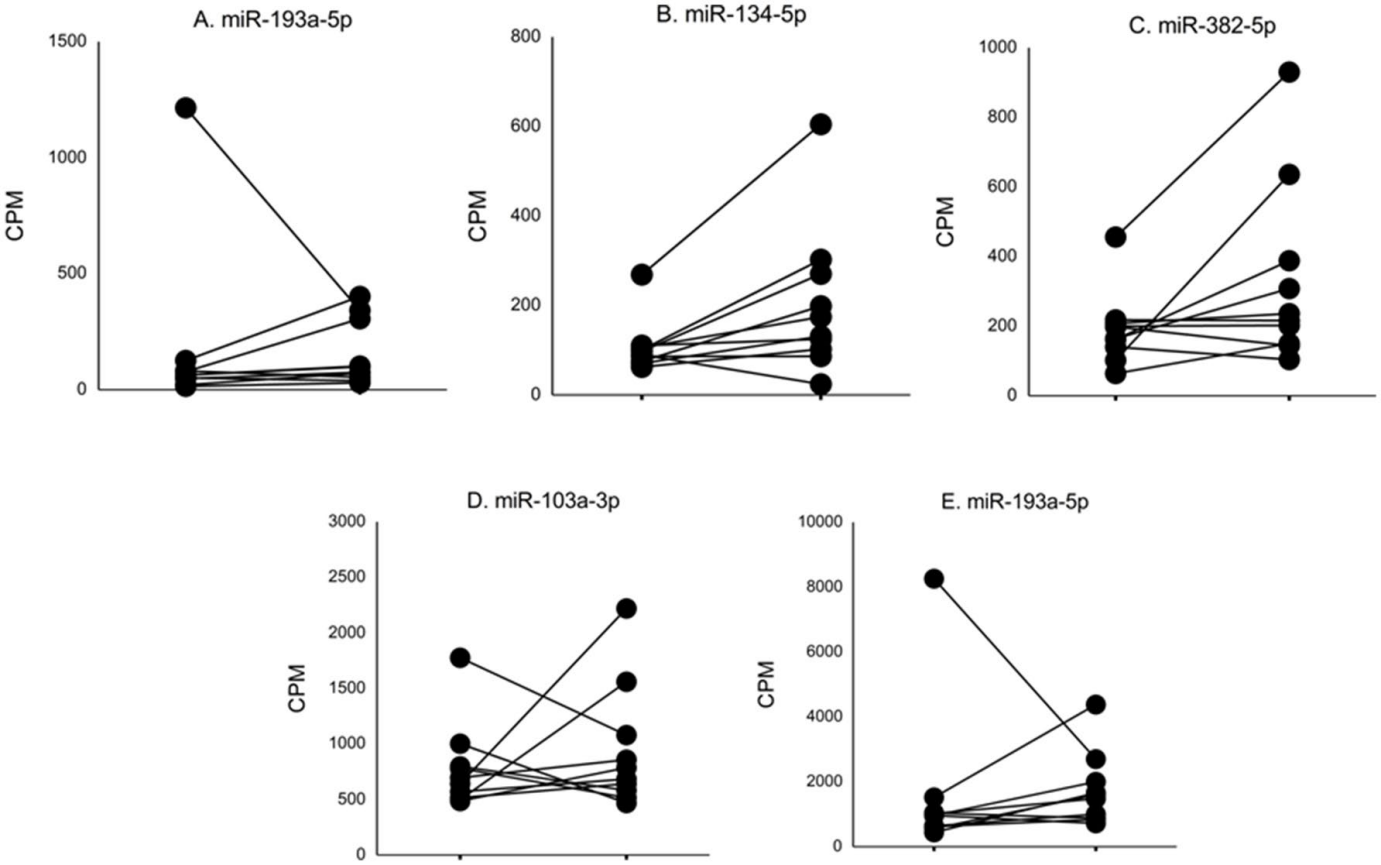
Changes in levels of five representative miRNA levels (miR-193a-5p, miR-134-5p, miR-382-5p, miR-1003a-3p, miR-378a-3p) at two time points in ASD subjects. These miRNAs revealed >2-fold DEs as compared to non-ASD control samples (**Figure 1**).

In nine ASD subjects, we obtained serum samples at two time points, and in one ASD subject, we obtained serum samples at three time points. This allowed us to examine whether serum levels of the miRNAs that differed from non-ASD controls remained at similar levels. The changes in serum levels of five representative miRNAs that revealed differences in the ASD subjects as compared to the non-ASD controls are shown in **Figure 2**. Although the number of samples is too small for statistical analysis, approximately 7 of the 10 subjects revealed stable miRNA levels.

Gene targets of miRNAs with differences in serum levels between ASD and non-ASD control sera were mainly associated with cell proliferation and differentiation, but pathways responsible for synaptic formation and plasticity (neurotrophin signaling and axon guidance) were also associated (summarized in **Table 7**). When the ASD group was analyzed as a whole, gene targets involving pathways specific for immune regulation were not identified.

### Clinical Characteristics and Serum miRNA Levels in the IL-1ß/IL-10 Ratio-Based ASD Subgroups

Consistent with our previous results, the IL-1ß/IL-10 ratio–based ASD subgroups revealed differences in both production of other cytokines and mitochondrial respiration (**Supplemental Table 2**). Clinical characteristics of the IL-1ß/IL-10 ratio–based ASD subgroups are consistent with our previous results; the low IL-1ß/IL-10 ratio ASD subgroup revealed a higher frequency of history of NFA (p < 0.05, Fisher’s exact test) (**Table 4**). There was no differences in frequency of gender, ASD severity, medication use (neuroleptics, ADHD meds, AEDs, and SSRIs) among the IL-1ß/IL-10 ratio–based ASD subgroups by chi-square test (p > 0.05). There was also no significant age difference between ASD subgroups and controls by Kruskal–Wallis test (p > 0.05).

**Table 4:** Demographics and clinical features in the IL-1ß/IL-10-based ASD subgroups.

	IL-1ß/IL-10 ratio–based ASD subgroups		
	High (N = 46)	Normal (N = 37)	Low (N = 22)
Age (yr) median (range) Age (yr) Mean ± SD	9.8 (2.5−21.5) 11.1 ± 5.6	11.4 (2.2−21.0) 11.5 ± 5.7	11.3 (4.3−19.8) 11.5 ± 4.5
Gender (M:F) Ethnicity	39:7 (84.5%:15.2%) AA 4. Asian 12, W 30.	30:7 (81.1%:18.9%) AA 1, Asian 8, W 27, mixed 1	19:3 (86.4%:13.6%) AA 2, Asian 3, W 17,
Cognitive activity (< 1^st^%)	33/46 (71.7%)	29/37 (78.4%)	16/22 (72.7%)
Disturbed sleep	14/46 (30.4%)	11/37 (29.7%)	11/22 (50.0%)
GI symptoms	29/46 (63.0%)	27/37 (73.0%)	17/22 (77.3%)
History of NFA^2^	26/46 (56.5%)	20/37 (54.1%)	18/22 (77.2%)^1^
Seizure disorders	4/46 (8.7%)	5/37 (13.5%)	4/22 (18.2%)
Specific antibody deficiency	8/46 (17.4%)	6/37 (16.2%)	6/22 (27.3%)
Allergic rhinitis	11/46 (23.9%)	8/37 (21.6%)	2/22 (9.1%)
Asthma	8/46 (17.4%)	4/37 (10.8%)	2/22 (9.1%)

^1^Low IL-1ß/IL-10 ratio ASD subgroup revealed a higher frequency of hx of NFA than the normal-ratio ASD subgroup (p = 0.048 by Fisher’s exact test).

^2^see **Table 1**.

**Figure 3:**
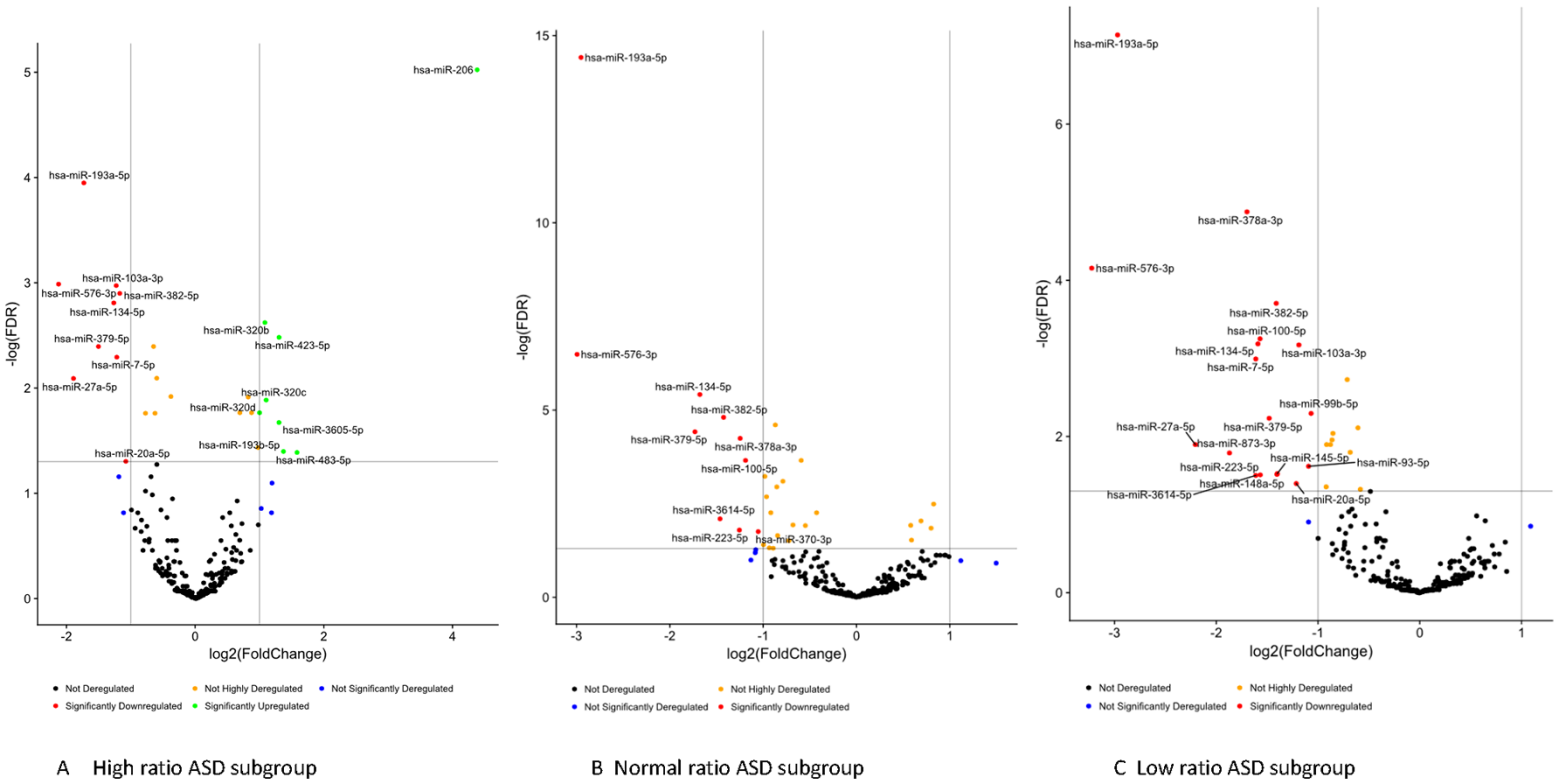
Volcano plots of differential expression of serum miRNAs in the IL-1ß/IL-10-based ASD subgroups (Panel **A**, **B**, and **C**) as compared to non-ASD controls.

Striking differences in expression of serum miRNA were found between the IL-1ß/IL-10 ratio–based ASD subgroups. Specifically, up-regulation of miRNA was observed only in the high-ratio ASD subgroup by DE analysis, as compared to non-ASD controls (**Table 5** and **Figure 3A**). In the normal- and low-ratio ASD subgroups, only downregulation of miRNA was observed, as compared to non-ASD controls (**Table 5** and **Figure 3B, C**). More miRNAs were down-regulated in the low-ratio ASD subgroup than the normal-ratio ASD subgroup (**Table 5**). Levels of 11 miRNAs differed among the ASD subgroups and the non-ASD controls when miRNA levels are expressed as TMM normalized readouts (CPM) by Kruskal–Wallis test (**Table 6**). Serum levels of miR-423-5p and miR-320b did not differ significantly when all the ASD samples combined were compared with non-ASD control samples. Since we found nominally significant differences of serum levels of several miRNAs with clinical variables (summarized in **Supplemental Table 1**), we also analyzed if these co-variant factors affect serum levels of these miRNAs among the ASD subgroups shown in **Table 6**. Covariance analysis rejected the effects of these variables on differences of serum levels of these miRNAs among the ASD subgroups (p < 0.05), except for miR-206 which revealed the effects of AEDs. Likewise, differences of serum levels of miRNAs shown in **Table 6** among the study groups were not affected by age by co-variance analysis (p < 0.05).

**Table 5:** Numbers of serum miRNAs that revealed >2-fold variations in expression in IL-1ß/IL-10-based ASD subgroups and total ASD samples as compared to non-ASD controls.

ASD sample groups	miRNA up-regulated^1^	miRNA down-regulated
High IL-1ß/IL-10 ratio (N = 48)	8	9
Normal IL-1ß/IL-10 ratio (N = 43)	0	10
Low IL-1ß/IL-10 ratio (N = 25)	0	18
All ASD samples (N = 116)^2^	4	14

^1^Numbers of miRNA more than two-fold difference by differential expression (DE) in IL-1ß/IL-10-based ASD subgroups vs. non-ASD controls (N = 35).

^2^Two and three samples were taken from nine and one select ASD subjects.

**Table 6:** Differences in miRNA levels in the IL-1ß/IL-10-based ASD subgroups.

	ASD IL-1ß/IL-10 ratio subgroups			Non-ASD Control (N = 35)	Statistics^1^
	High (N = 48)	Normal (N = 43)	Low (N = 25)		
miR-206^2^	719.6 ± 4,634.3	79.7 ± 124.8	25.1 ± 26.1	34.5 ± 73.8	0.0063
miR-193a-5p^3^	212.1 ± 245.0	106.2 ± 84.0	110.9 ± 104.7	817.0 ± 1,325.9	0.0001
miR-379-5p	79.2 ± 90.4	70.1 ± 50.6	85.6 ± 57.9	223.9 ± 260.5	0.0108
miR-134-5p	15.7 ± 156.0	137.2 ± 159.9	147.5 ± 123.8	397.4 ± 486.7	0.0012
miR-184	31.5 ± 58.0	44.0 ± 120.4	14.9 ± 23.4	124 ± 27.5	0.0038
miR-382-5p	206.8 ± 178.7	186.7 ± 151.1	195.9 ± 105.6	484.1 ± 428.6	0.0005
miR-103a-3p	578.5 ± 349.3	681.3 ± 349.1	616.0 ± 158.2	1,342.6 ± 1,342.1	0.0261
miR-378a-3p	1,989.9 ± 1,904.0	1,526.5 ± 1,725.0	1,135.4 ± 680.6	3,491.2 ± 3,865.8	0.0106
miR-3614-5p	51.5 ± 76.1	26.2 ± 46.7	25.8 ± 32.8	75.3 ± 103.6	0.0311
miR-423-5p	66,514.8 ± 75,815	46,085.5 ± 52,774	42,478.9 ± 29,528.9	27,956.6 ± 26,599	0.0074
miR-320b	715.0 ± 460.4	547.4 ± 458.5	519.7 ± 247.9	368.0 ± 249.9	0.0116

^1^Kruskal–Wallis test was used for assessing differences. Serum miRNA levels were expressed TMM normalized read counts (CPM) ± SD as detailed in the methods section.

^2^Higher miRNA levels in the normal-ratio ASD subgroup as compared to the low-ratio ASD subgroup by Mann–Whitney test (p < 0.05).

^3^Higher miRNA levels in the high-ratio ASD subgroup as compared to the normal-ratio ASD subgroup (p < 0.05 by Mann–Whitney test).

### Pathways Enriched by Target Genes by Serum miRNAs in the ASD Subgroups

Gene targets of miRNAs that differed between IL-1ß/IL-10 ratio ASD subgroups and non-ASD controls were also examined. Targeted genes by each miRNA in this analysis were shown in the excel spread sheet as a supplement. Then, we determined whether the genes in a specific pathway are enriched (over-represented) by genes targeted by miRNAs that revealed >2-fold DEs in the ASD subgroups, as compared to non-ASD controls. **Table 7** shows the pathways that revealed enrichment in each ASD subgroup, or in all the ASD samples combined (Bonferroni test, p < 0.001). The high- and low-ratio ASD subgroups revealed that miRNA targeted genes enriched are associated with pathways critical in innate immune responses (**Table 7**). In the high-ratio ASD subgroup, miRNA targeted genes were enriched in pathways with intracellular trafficking secretion, mTOR, and insulin signaling pathways. In the low-ratio ASD subgroup, targeted genes were enriched in pathways of biquitin-mediated proteolysis and adherens junction pathways. In both the high- and low-ratio ASD subgroups, miRNA target genes are enriched in pathways that are crucial to monocyte/macrophage functions (endocytosis, focal adhesion, TGF-ß-signaling, and WNT signaling). Subgroup-specific associations were not observed in the normal-ratio ASD subgroup (**Table 7**).

**Table 7:** Pathways enriched by miRNA target genes in the IL-1ß/IL-10-based ASD subgroups.

IL-1ß/IL-10 ratio	Pathways revealed enrichment by miRNA target genes^1^
High-ratio ASD subgroup only	Renal cell carcinoma, melanoma *Intracellular trafficking and secretion* *^2^* *mTOR signaling pathway* *^2^* *, insulin signaling pathway* *^2^*
Low-ratio ASD subgroup only	*Ubiquitin-mediated proteolysis* *^2^* *, adherens junction* *^2^*
Both high- and low-ratio ASD subgroups	Endocytosis^2^, focal adhesion^2^, TGF-ß signaling pathway^2^ WNT signaling pathway^2^ Chronic myeloid leukemia, pancreatic cancer, oocyte meiosis Colorectal cancer
Normal-ratio ASD subgroup only^4^	None
All ASD samples combined	Pathways in cancer, prostate cancer, non-small cell lung cancer, glioma, ErB signaling pathway *Neurotrophin signaling pathway* *^3^* *, axon guidance* *^3^* *MAPK signaling pathway* *^2^*

^1^Revealed enrichment by target genes by miRNAs that revealed >2-fold DEs as compared to non-ASD controls by Bonferroni test (p < 0.001).

^2^Pathways associated with key immune functions and metabolic regulation

^3^Pathways associated with synaptic formation and neuronal plasticity.

^4^Normal IL-1ß/IL-10 ratio ASD groups—no subgroup specific pathway was found in this group. Pathways associated are the same as shown in all ASD samples.

### Correlations Between Serum miRNA With IL-1ß/IL-10 Ratios and Monocyte Cytokine Profiles

We also assessed the linear relationship between serum miRNA and IL-1ß/IL-10 ratios produced by PBMo and monocyte cytokine profiles, since immunological pathways targeted by miRNAs differed between the IL-1ß/IL-10-based ASD subgroups. Cellular samples were obtained simultaneously at the time of serum collection.

Our results revealed significant differences in correlations between IL-1ß/IL-10 ratios and serum miRNA levels among the ASD subgroups, as compared to non-ASD controls. We also found differences in linear correlations between serum miRNA levels and PBMo cytokine profiles in the IL-1ß/IL-10-based ASD subgroups. This was most significant for TNF-α, IL-10, and CCL2. These results were summarized in **Tables 8**–**11**. In the low-ratio ASD subgroup, a majority of miRNAs that revealed significant correlations with these monocyte cytokine parameters are down-regulated as compared to non-ASD controls.

**Table 8:** Correlations between IL-1ß/IL-10 ratios and serum miRNA levels in IL-ß/IL-10-based ASD subgroups and non-ASD controls.

Groups and culture conditions	miRNA revealed correlations with IL-1ß/IL-10 ratios	
Non-ASD controls (N = 35)	Positive correlation	Negative correlation
Medium	No significant correlation	No significant correlation
LPS	576-3p^2^ (0.3843, p < 0.05)^1^ 134-5p^2^ (0.3426, p < 0.05) 100-5p (0.4706, p < 0.005) 99b-5p (0.3877, p < 0.05)	27a-5p^2^ (−0.4516, p < 0.001) 223-5p^2^ (−0.4354, p < 0.01) 103a-5p^2^ (−0.3479, p < 0.05) 433-3p^2^ (0.356, p < 0.05) 20a-5p (−0.4006, p < 0.05)
Zymosan	423-5p (0.4854, p < 0.005) 320d (0.4403, p < 0.01) 370-3p (0.37, p < 0.05)	27a-5p^2^ (−0.5993, p < 0.0001) 574-3p^2^ (−0.317, p < 0.05) 223-5p^2^ (−0.4812, p < 0.005) 3,614-5p^2^ (−0.4107, p < 0.05) 20a-5p (−0.3354, p < 0.05)
CL097	576-3p^2^ (0.5459, p < 0.001) 193a-5p^2^ (0.416, p < 0.05) 379-5p^2^ (0.3378, p < 0.05) 134-5p^2^ (0.5246, p < 0.005) 382-5p^2^ (0.5028, p < 0.005) 378a-3p^2^ (0.4675, p < 0.005) 873-3p^2^ (0.3532, p < 0.05) 483-5p (0.5448, p < 0.001) 320b (0.386, p < 0.05) 100-5p (0.4103, p < 0.05)	4,732-5p^2^ (−0.4277, p < 0.05) 193b-5p (−0.3687, p < 0.05)
ASD high-ratio subgroup (N = 48)	**Positive correlation**	**Negative correlation**
Medium	576-3p^2^ (0.3842, p < 0.05) 378-3p^2^ (0.3251, p < 0.05) 873-3p^2^ (0.526, p < 0.001) 100-5p (0.3921, p < 0.05)	
LPS	No significant correlation	No significant correlation
Zymosan	193a-5p^2^ (0.4078, p < 0.05) 379-5p^2^ (0.4514, p < 0.005) 134-32^2^ (0.554, p < 0.0005) 382-5p^2^ (0.4565, p < 0.005) 223-5p^2^ (0.3686, p < 0.05) 378a-3p^2^ (0.4056, p < 0.05) 433-3p^2^ (0.5614, p < 0.005)	423-5p^2^ (−0.3704, p < 0.05)
CL097	No significant correlation	423-5p (−0.4065, p < 0.05)
ASD normal-ratio subgroup (N = 43)	**Positive correlation**	**Negative correlation**
Medium	423-5p (0.3226, p < 0.05)	No significant correlation
LPS	No significant correlation	No significant correlation
Zymosan	4,732-5p^2^ (0.3739, p < 0.05) 93-5p (0.3363, p < 0.05)	No significant correlation
CL097	483-5p (0.3499, p < 0.05) 320b (0.3682 (p < 0.05)	3,614-5p^2^ (−0.3432, p < 0.05)
ASD low-ratio subgroup (N = 25)	**Positive correlation**	**Negative correlation**
Medium	873-3p^2^ (0.4661, p < 0.05)	370-3p (−0.4113, p < 0.05)
LPS	No significant correlation	No significant correlation
Zymosan	320d (0.4478, p < 0.05)	No significant correlation
CL097	379-5p^2^ (0.5461, p < 0.01) 99b-5p (0.5496, p < 0.01)	No significant correlation

^1^All miRNAs revealed significant positive or negative correlations were shown. Values of correlation coefficient and p-values were shown in ( ).

^2^miRNAs revealed significant DEs in all the ASD samples combined as compared to non-ASD controls.

**Table 9:** Correlations between serum miRNA levels and IL-10 levels produced by PBMo in IL-1ß/IL-10 ratio–based ASD subgroups and non-ASD controls.

Groups and culture conditions	miRNAs revealed correlations with IL-10 levels	
Non-ASD controls (N = 35)	Positive correlation	Negative correlation
Medium	320d (−0.349, p < 0.05)^1^	379-5p^2^ (−0.4574, p < 0.01) 134-5p^2^ (−0.4068, p < 0.05) 382-5p^2^ (−0.4145, p < 0.05) 433-3p^2^ (−0.3704, p < 0.05)
LPS	574-3p^2^ (0.3793, p < 0.05)	379-5p^2^ (−0.4703, p < 0.005) 134-5p^2^ (−0.3412, p < 0.05) 382-5p^2^ (−0.3417, p < 0.05)
Zymosan	No significant correlation	483-5p (−0.3809, p < 0.05)
CL097	No significant correlation	483-5p (−0.3992, p < 0.05) 320d (−0.4011, p < 0.05)
ASD high-ratio subgroup (N = 48)	**Positive correlation**	**Negative correlation**
Medium	No significant correlation	No significant correlation
LPS	No significant correlation	433-3p^2^ (−0.366, p < 0.05)
Zymosan	No significant correlation	223-5p^2^ (−0.4188, p < 0.01) 93-5p (−0.4218, p < 0.01)
CL097	No significant correlation	No significant correlation
ASD normal-ratio subgroup (N = 43)	**Positive correlation**	**Negative correlation**
Medium	574-3p^2^ (0.3646, p < 0.05)	No significant correlation
LPS	No significant correlation	7-5p^2^ (−0.3571, p < 0.05) 4732-5p (−0.3152, p < 0.05)
Zymosan	103a-3p^2^ (0.3767, p < 0.05)	483-5p (−0.3161, p < 0.05) 100-5p (−0.3501, p < 0.05)
CL097	574-3p^2^ (0.3268 p < 0.05) 93-5p (0.3134, p < 0.05)	382-5p^2^ (−0.3266, p < 0.05)
ASD low-ratio subgroup (N = 25)	**Positive correlation**	**Negative correlation**
Medium	No significant correlation	No significant correlation
LPS	No significant correlation	93-5p (0.500, p < 0.05)
Zymosan	No significant correlation	3,614-5p^2^ (−0.48, p < 0.05)
CL097	184^2^ (0.436, p < 0.05)	No significant correlation

^1^All miRNAs revealed significant positive or negative correlations were shown. Values of correlation coefficient and p-values were shown in ( ).

^2^miRNAs revealed significant DEs in all the ASD samples combined as compared to non-ASD controls.

**Table 10:** Correlations between serum miRNA levels and TNF-α levels produced by PBMo in IL-1ß/IL-10-based ASD subgroups and non-ASD controls.

Groups and culture conditions	miRNAs revealed correlations with IL-10 levels			
Groups and culture conditions		miRNAs revealed correlations with IL-10 levels		
Non-ASD (N = 35)		Positive correlation		Negative correlation
Non-ASD controls (N = 35)	**Positive correlation**		**Negative correlation**	
Medium	576-3p^2^ (0.5787, p < 0.0005)^1^ 193a-5p^2^ (0.4173, p < 0.05) 134-5p^2^ (0.3954, p < 0.05) 382-5p^2^ (0.3706, p < 0.05) 7-5p^2^ (0.4124, p < 0.05) 378-5p^2^ (0.4869, p < 0.005)		184^2^ (−0.5056, p < 0.005) 4,732-5p^2^ (−0.5615, p < 0.0005) 193b-5p^2^ (−0.5006, p < 0.005) 423-5p (−0.3845, p < 0.05) 483-5p (−0.3857, p < 0.05)	
LPS	576-3p^2^ (0.668, p < 0.0001) 193a-5p^2^ (0.3714, p < 0.05) 134-5p^2^ (0.3541, p < 0.05) 382-5p^2^ (0.3456, p < 0.05) 7-5p^2^ (0.3856, p < 0.05) 378-5p^2^ (0.4247, p < 0.05) 100-5p (0.3393, p < 0.05)		4,732-5p^2^ (−0.5304, p < 0.001) 193b-5p^2^ (−0.4634, p < 0.01) 423-5p (−0.3534, p < 0.05) 483-5p (−0.4097, p < 0.05)	
Zymosan	576-3p^2^ (0.7227, p < 0.0001) 193a-5p^2^ (0.6793, p < 0.0001) 379-5p^2^ (0.4279, p < 0.05) 134-5p^2^ (0.5231, p < 0.005) 382-5p^2^ (0.6139, p = 0.0001) 7-5p^2^ (0.6866, p < 0.0001) 103a-5p^2^ (0.4311, p < 0.01) 378-5p^2^ (0.7478, p < 0.0001) 3,614-5p^2^ (0.399, p < 0.05) 20a-5p (0.3531, p < 0.05) 93-5p (0.411, p < 0.05)		206^2^ (−0.4448, p < 0.01) 184^2^ (−0.3349, p < 0.05) 4,732-5p^2^ (−0.7519, p < 0.0001) 193b-5p^2^ (−0.6518, p < 0.0001) 423-5p (−0.6893, p < 0.0001) 483-5p (−0.567, p < 0.0005) 320b (−0.5776, p < 0.005) 320d (−0.3751, p < 0.05)	
CL097	576-3p^2^ (0.6937, p < 0.0001) 193a-5p^2^ (0.6056, p < 0.0005) 379-5p^2^ (0.4441, p < 0.05) 134-5p^2^ (0.5038, p < 0.005) 382-5p^2^ (0.5621, p < 0.001) 7-5p^2^ (0.6081, p = 0.0001) 103a-5p^2^ (0.6575, p < 0.0001) 3,614-5p^2^ (0.4008, p < 0.05) 20a-5p (0.3694, p < 0.05) 93-5p (0.4606, p < 0.01)		206^2^ (−0.4169, p < 0.05) 4,732-5p^2^ (−0.7037, p < 0.0001) 193b-5p^2^ (−0.6597, p < 0.0001) 423-5p (−0.6694, p < 0.0001) 483-5p (−0.5947, p < 0.0005) 320b (−0.6095, p = 0.0001) 320d (−0.3801, p < 0.05)	
ASD high-ratio subgroup (N = 48)	**Positive correlation**		**Negative correlation**	
Medium	93-3p (0.3744, p < 0.05)		873-3p^2^ (−0.3403, p < 0.05)	
LPS	No significant correlation		873-3p^2^ (−0.3537, p < 0.05)	
Zymosan	No significant correlation		No significant correlation	
CL097	No significant correlation		423-5p (−0.4038, p < 0.05)	
ASD normal-ratio subgroup (N = 43)	**Positive correlation**		**Negative correlation**	
Medium	No significant correlation		No significant correlation	
LPS	No significant correlation		No significant correlation	
Zymosan	193a-5p^2^ (0.3847, p < 0.05) 223-5p^2^ (0.3434, p < 0.05) 103a-3p^2^ (0.3107, p < 0.05) 378a-3p^2^ (0.3045, p < 0.05) 93-5p (0.391, p < 0.01)		382-5p^2^ (−0.3784, p < 0.05) 370-3p (−0.3236, p < 0.05)	
CL097	No significant correlation		No significant correlation	
ASD low-ratio subgroup (N = 25)	**Positive correlation**		**Negative correlation**	
Medium	193a-5p^2^ (0.5736, p < 0.005)		No significant correlation	
LPS	193a-5p^2^ (0.5494, p < 0.01) 184^2^ (0.4449, p < 0.05) 193b-5p^2^ (0.4131, p < 0.05)		No significant correlation	
Zymosan	193a-5p^2^ (0.407, p < 0.05) 134-5p^2^ (0.5487, p < 0.01) 382-5p^2^ (0.5496, p < 0.01) 433-3p^2^ (0.4243, p < 0.05)		No significant correlation	
CL097	379-5p^2^ (0.4143, p < 0.05) 99b-5p (0.4294, p < 0.05)		206^2^ (−0.4696, p < 0.05)	
Medium		223-3p^2^ (0.4021, p < 0.05) 7-5p^2^ (0.3516, p < 0.05) 378a-3p^2^ (0.356, p < 0.05) 20a-5p (0.3751, p < 0.05) 93-5p (0.3724, p < 0.05)		423-5p (−0.3457, p < 0.05) 483-5p (−0.3554, p < 0.05) 320b (−0.4173, p < 0.05) 320d (−0.4859, p < 0.05)
LPS		27a-5p^2^ (0.4294, p = 0.01) 223-3p^2^ (0.4503, p < 0.01) 103a-3p^2^ (0.4007, p < 0.05) 433-3p^2^ (0.4608, p < 0.01) 20a-5p (0.5278, p < 0.005)		573-3p^2^ (−0.435, p < 0.01) 320d (−0.429, p < 0.05) 100-5p (−0.7611, p < 0.0001) 99b-5p (−0.5002, p < 0.005)
Zymosan		27a-5p^2^ (0.3892, p < 0.05) 223-3p^2^ (0.3465, p < 0.05) 433-3p^2^ (0.3686, p < 0.05) 20a-5p (0.4367, p < 0.01)		573-3p^2^ (−0.3863, p < 0.05) 4,732-5p^2^ (−0.4074, p < 0.05) 320d (−0.4125, p < 0.05) 100-5p (−0.6933, p < 0.0001) 99b-5p (−0.5254, p < 0.005)
CL097		4,732-5p^2^ (0.4445, p < 0.01) 193b-5p^2^ (0.3763, p < 0.05) 433-3p^2^ (0.35, p < 0.05)		573-3p^2^ (−0.5331, p < 0.001) 193a-5p^2^ (−0.4203, p < 0.05) 378a-3p^2^ (−0.3885, p < 0.05) 873-3p^2^ (−0.3885, p < 0.05) 99b-5p (−0.3562, p < 0.05)
ASD high-ratio subgroup (N = 48)		**Positive correlation**		**Negative correlation**
Medium		No significant correlation		No significant correlation
LPS		99b-5p (0.4161, p < 0.01)		576-3p^2^ (−0.3249, p < 0.05) 7-5p^2^ (−0.4198, p < 0.01) 20a-5p (−0.4546, p < 0.005)
Zymosan		574-3p^2^ (0.501, p < 0.005) 193a-5p^2^ (0.3715, p < 0.05)		379-5p^2^ (−0.4006, p < 0.05 134-5p^2^ (−0.3424, p < 0.05) 433-3p^2^ (−0.371, p < 0.05)
CL097		206^2^ (0.3227, p < 0.05) 99b-5p (0.382, p < 0.05)		7-5p^2^ (−0.4179, p < 0.01) 20a-5p (−0.3726, p < 0.05) 93-5p (−0.5227, p < 0.001)
ASD normal-ratio subgroup (N = 43)		**Positive correlation**		**Negative correlation**
Medium		378a-3p^2^ (0.3477, p < 0.05)		433-3p^2^ (−0.4146, p < 0.01)
LPS		193a-5p^2^ (0.3269, p < 0.05)		433-3p^2^ (−0.3285, p < 0.05)
Zymosan		423-5p (0.3136, p < 0.05) 100-5p (0.4193, p < 0.01)		No significant correlation
CL097		7-5p^2^ (0.3593, p < 0.05) 378a-3p^2^ (0.4558, p < 0.005)		433-3p^2^ (−0.3611, p < 0.05)
ASD low-ratio subgroup (N = 25)		**Positive correlation**		**Negative correlation**
Medium		193a-5p^2^ (0.4626, p < 0.05)		No significant correlation
LPS		No significant correlation		223-5p^2^ (−0.4078, p < 0.05)
Zymosan		576-3p^2^ (0.4983, p < 0.05) 320d (0.4365, p < 0.05) 93-5p (0.473, p < 0.05)		No significant correlation
CL097		576-3p^2^ (0.5252, p < 0.01)		No significant correlation

^1^All miRNAs revealed significant positive or negative correlations were shown. Values of correlation coefficient and p-values were shown in ( ).

^2^miRNAs revealed significant DEs in all the ASD samples combined as compared to non-ASD controls.

^1^All miRNAs revealed significant positive or negative correlations were shown. Values of correlation coefficient and p-values were shown in ( ).

^2^miRNAs revealed significant DEs in all the ASD samples combined as compared to non-ASD controls.

**Table 11:** Correlations between serum miRNA levels and CCL2 levels produced by PBMo in the IL-1ß/IL-10-based ASD subgroups and non-ASD controls.

Groups and culture conditions	miRNAs revealed correlations with IL-10 levels	
Non-ASD (N = 35)	Positive correlation	Negative correlation
Medium	223-3p^2^ (0.4021, p < 0.05) 7-5p^2^ (0.3516, p < 0.05) 378a-3p^2^ (0.356, p < 0.05) 20a-5p (0.3751, p < 0.05) 93-5p (0.3724, p < 0.05)	423-5p (−0.3457, p < 0.05) 483-5p (−0.3554, p < 0.05) 320b (−0.4173, p < 0.05) 320d (−0.4859, p < 0.05)
LPS	27a-5p^2^ (0.4294, p = 0.01) 223-3p^2^ (0.4503, p < 0.01) 103a-3p^2^ (0.4007, p < 0.05) 433-3p^2^ (0.4608, p < 0.01) 20a-5p (0.5278, p < 0.005)	573-3p^2^ (−0.435, p < 0.01) 320d (−0.429, p < 0.05) 100-5p (−0.7611, p < 0.0001) 99b-5p (−0.5002, p < 0.005)
Zymosan	27a-5p^2^ (0.3892, p < 0.05) 223-3p^2^ (0.3465, p < 0.05) 433-3p^2^ (0.3686, p < 0.05) 20a-5p (0.4367, p < 0.01)	573-3p^2^ (−0.3863, p < 0.05) 4,732-5p^2^ (−0.4074, p < 0.05) 320d (−0.4125, p < 0.05) 100-5p (−0.6933, p < 0.0001) 99b-5p (−0.5254, p < 0.005)
CL097	4,732-5p^2^ (0.4445, p < 0.01) 193b-5p^2^ (0.3763, p < 0.05) 433-3p^2^ (0.35, p < 0.05)	573-3p^2^ (−0.5331, p < 0.001) 193a-5p^2^ (−0.4203, p < 0.05) 378a-3p^2^ (−0.3885, p < 0.05) 873-3p^2^ (−0.3885, p < 0.05) 99b-5p (−0.3562, p < 0.05)
ASD high-ratio subgroup (N = 48)	**Positive correlation**	**Negative correlation**
Medium	No significant correlation	No significant correlation
LPS	99b-5p (0.4161, p < 0.01)	576-3p^2^ (−0.3249, p < 0.05) 7-5p^2^ (−0.4198, p < 0.01) 20a-5p (−0.4546, p < 0.005)
Zymosan	574-3p^2^ (0.501, p < 0.005) 193a-5p^2^ (0.3715, p < 0.05)	379-5p^2^ (−0.4006, p < 0.05 134-5p^2^ (−0.3424, p < 0.05) 433-3p^2^ (−0.371, p < 0.05)
CL097	206^2^ (0.3227, p < 0.05) 99b-5p (0.382, p < 0.05)	7-5p^2^ (−0.4179, p < 0.01) 20a-5p (−0.3726, p < 0.05) 93-5p (−0.5227, p < 0.001)
ASD normal-ratio subgroup (N = 43)	**Positive correlation**	**Negative correlation**
Medium	378a-3p^2^ (0.3477, p < 0.05)	433-3p^2^ (−0.4146, p < 0.01)
LPS	193a-5p^2^ (0.3269, p < 0.05)	433-3p^2^ (−0.3285, p < 0.05)
Zymosan	423-5p (0.3136, p < 0.05) 100-5p (0.4193, p < 0.01)	No significant correlation
CL097	7-5p^2^ (0.3593, p < 0.05) 378a-3p^2^ (0.4558, p < 0.005)	433-3p^2^ (−0.3611, p < 0.05)
ASD low-ratio subgroup (N = 25)	**Positive correlation**	**Negative correlation**
Medium	193a-5p^2^ (0.4626, p < 0.05)	No significant correlation
LPS	No significant correlation	223-5p^2^ (−0.4078, p < 0.05)
Zymosan	576-3p^2^ (0.4983, p < 0.05) 320d (0.4365, p < 0.05) 93-5p (0.473, p < 0.05)	No significant correlation
CL097	576-3p^2^ (0.5252, p < 0.01)	No significant correlation

^1^All miRNAs revealed significant positive or negative correlations were shown. Values of correlation coefficient and p-values were shown in ( ).

^2^miRNAs revealed significant DEs in all the ASD samples combined as compared to non-ASD controls.

### Correlations Between Serum miRNA Levels and Mitochondrial Respiration by PBMCs in the IL-1ß/IL-10-Based ASD Subgroups

We also assessed possible correlations between serum miRNA levels and mitochondrial respiration by PBMCs to test our hypotheses that serum miRNAs reflect mitochondrial respiration. Correlations between mitochondrial parameters and serum miRNA levels also differed across the IL-1ß/IL-10-based ASD subgroups and non-ASD controls (**Table 12**). Predominant negative correlations were observed between the parameters of mitochondrial respiration (PLR, ALR, MRC, and RC) and serum miRNA levels in non-ASD controls, and some of them revealed p values <0.005 (**Table 12**). In contrast, such correlations were less evident in the ASD subgroups. The exception was negative correlations between PLR/ALR and serum levels of several miRNAs in the normal-ratio ASD subgroup (**Table 12**).

**Table 12:** Correlations between miRNA levels and mitochondrial respiration by PBMCs.

Groups and OCR parameters	Serum miRNAs revealed correlations with mitochondrial respiration parameters	
Non-ASD controls (N = 35)	Positive correlation	Negative correlation
PLR	370-3p (0.557, p < 0.005) 99b-5p (0.5127, p < 0.005)	193a-5p^2^ (−0.4417, p < 0.05) 223-5p^2^ (−0.5124, p < 0.005) 20a-5p (−0.4136, p < 0.05)
ALR	423-5p (0.5767, p < 0.01) 370-3p (0.4186, p < 0.005)	576-3p^2^ (−0.4115, p < 0.05) 193a-5p^2^ (−0.6035, p < 0.0005) 27a-5p^2^ (−0.5413, p < 0.005) 223-5p^2^ (−0.637, p < 0.0001) 7-5p^2^ (−0.3563, p < 0.05) 103a-3p^2^ (−0.3752, p < 0.05) 378a-3p^2^ (−0.513, p < 0.005) 3,614-5p^2^ (−0.523, p < 0.005) 93-5p (−0.4318, p < 0.05)
MRC	423-5p (0.5246, p < 0.005) 370-3p (0.3785, p < 0.05)	576-3p^2^ (−0.375, p < 0.05) 193a-5p^2^ (−0.4962, p < 0.005) 27a-5p^2^ (−0.4451, p < 0.05) 223-5p^2^ (−0.4442, p < 0.05) 7-5p^2^ (−0.3567, p < 0.05) 378a-3p^2^ (−0.448 (p < 0.05) 3,614-5p^2^ (−0.5119, p < 0.005) 93-5p (−0.4222, p < 0.05)
RC	No significant correlation	3,614-5p^2^ (−0.3683, p < 0.05)
ASD high-ratio subgroup (N = 48)	**Positive correlation**	**Negative correlation**
PLR	No significant correlation	No significant correlation
ALR	No significant correlation	193a-5p^2^ (−0.3923, p < 0.05) 378a-3p^2^ (−0.4223, p < 0.01)
MRC	No significant correlation	No significant correlation
RC	No significant correlation	No significant correlation
ASD normal-ratio subgroup (N = 43)	**Positive correlation**	**Negative correlation**
PLR	574-3p^2^ (0.3506, p < 0.05)	7-5p^2^(−0.4181, p < 0.01) 378a-3p^2^ (−0.3195, p < 0.05) 4,732-5p^2^ (−0.3318, p < 0.05)
ALR	No significant correlation	193a-5p^2^ (−0.341, p < 0.05) 378a-3p^2^ (−0.3604, p < 0.05)
MRC	No significant correlation	No significant correlation
RC	No significant correlation	No significant correlation
ASD low-ratio subgroup (N = 25)	**Positive correlation**	**Negative correlation**
PLR	100-5p (−0.5534, p < 0.05)	No significant correlation
ALR	No significant correlation	No significant correlation
MRC	No significant correlation	No significant correlation
RC	No significant correlation	382-5p^2^ (−0.4719, p < 0.05)

^1^All miRNAs revealed significant positive or negative correlations were shown. Values of correlation coefficient and p-values were shown in ( ).

^2^miRNAs revealed significant DEs in all the ASD samples combined as compared to non-ASD controls.

Detailed results of correlations between serum miRNA levels and IL-1ß/IL-10 ratios are shown in **Table 8**. In non-ASD controls, positive and negative correlations were noted between serum levels of several miRNAs and IL-1ß/IL-10 ratios in the LPS, zymosan, and CL097 stimulated cultures. Five, one, and one serum miRNA levels had positive correlations with p < 0.005 in CL097, zymosan, and LPS stimulated cultures, respectively (**Table 8**). Fewer correlations were observed in the IL-1ß/IL-10-based ASD subgroups, and observed correlations were mostly positive correlations (**Table 8**). Only the high IL-1ß/IL-10 ratio ASD subgroup revealed positive correlations (p < 0.005) in four miRNAs and one miRNA between miRNA serum levels and the IL-1ß/IL-10 ratios produced under zymosan and LPS-stimulated cultures, respectively (**Table 8**). These miRNAs differed from non-ASD controls when compared the ASD samples as a whole and not affected by any clinical co-variables.

Correlations between IL-10 production by PBMo and serum miRNA levels revealed quite different results. Specifically, negative correlations were mainly observed between IL-10 production by PBMo and serum miRNA levels in the non-ASD controls, and these correlations were observed in most culture conditions tested (**Table 9**). The high- and low-ratio ASD subgroups revealed much less correlations between IL-10 production and serum levels of miRNAs (**Table 9**). The normal-ratio ASD subgroup revealed positive and negative correlations in several miRNAs between IL-10 production and serum miRNA levels (**Table 9**).

As observed with the IL-1ß/IL-10 ratios, significant positive correlations were observed between TNF-α production by PBMo and serum levels of multiple miRNAs in non-ASD controls with many of these correlations having p values <0.005 (**Table 10**). These miRNAs revealed significant correlations in most culture conditions tested (**Table 10**). In contrast, in the high-ratio ASD subgroup, a positive correlation was observed only in miR-93-3p (p < 0.05) between serum miRNA levels and TNF-α production by PBMo. In the normal- and low-ratio ASD subgroups, mainly positive correlations were found in less numbers of miRNAs (**Table 10**).

Correlations between CCL2 production by PBMo and serum miRNA levels also differed across the ASD subgroups and non-ASD controls (**Table 11**). In the non-ASD controls, correlations between these two parameters were found in multiple miRNAs, and these miRNAs revealed associations in multiple culture conditions (**Table 11**). In the high and normal IL-1ß/IL-10 ratio ASD subgroups, there were less correlations between CCL2 production and serum miRNA levels (**Table 11**). Least correlations between CCL2 production and serum miRNA levels were found in the low-ratio ASD subgroup, and these are mostly positive correlations.

## Discussion

Our results revealed alterations in serum miRNA levels in the IL-1ß/IL-10-based ASD subgroups. We also found altered correlations between serum miRNA levels and monocyte cytokine profiles and mitochondrial respiration across the IL-1ß/IL-10-based ASD subgroups. These results are consistent with our finding that genes targeted by miRNAs that showed differences in serum levels from non-ASD controls are associated with pathways, crucial for monocyte functions and metabolic regulation in the high and low IL-1ß/IL-10 ratio ASD subgroups. Although further studies are required, our results indicate that serum miRNA levels may be promising candidate biomarkers for screening changes in innate immune responses in ASD.

ASD is a complex developmental disorder. Converging effects of multiple genetic and environmental factors appear to create the basis for the pathogenesis of ASD. Mounting evidence from recent research indicates a role for chronic inflammation in the etiology and ongoing pathophysiology of ASD (2–4). Considering the variable clinical features and co-morbid conditions observed in ASD patients, it is unlikely that chronic inflammation universally affects all ASD subjects. However, currently, no easily accessible objective biomarkers exist for screening whether inflammation plays a role in each individual with ASD. Identification of such objective biomarker(s) will be tremendously helpful in the identification and treatment of individuals with ASD who have a component of chronic inflammation.

When assessing the role of inflammation, findings from ASD animal models have been very informative and intriguing. In the maternal immune activation (MIA) model, one of the most studied animal models of ASD, sterile inflammation is induced during pregnancy through activation of innate immunity (34, 35). Offspring exposed to MIA have lasting changes in cognitive activity and behavioral symptoms into their adulthood (35–37). Lasting effects of MIA have also been reported in both innate and adaptive immune responses (36).

As opposed to adaptive immunity, innate immune responses were thought to be non-specific and transient, lacking memory. However, recent discoveries of lasting effects of immunological challenges on innate immunity drastically changed our concept of innate immune memory. It was found that innate immune cells have long-term memory following an initial immune challenge, resulting in exaggerated responses to a secondary stimulus (38, 39). This is accomplished by the reprograming of both the metabolic and epigenetic pathways (38, 39). As a result, in contrast to adaptive immunity, the secondary stimulus can be different than the first stimulus. The term “trained immunity (TI)” is now being used, when referring to such changes. TI has been found to occur in both mature and immature myeloid progenitor cells (40–43), leading to lasting effects. Prominent metabolic changes that have been noted to occur with TI include a shift in energy metabolism from oxidative phosphorylation to aerobic glycolysis and an increase in cholesterol synthesis and glutaminolysis (43), which likely leads to changes in mitochondrial respiration.

Various immunological challenges, including microbial infection, changes in microbiota, and diets, as well as other environmental factors, are implicated with the induction of TI (38, 41–43). The key cytokines involved in this process may vary for each rodent model of TI. For example, in the models of ß-glucan-induced TI, the importance of IL-1ß was stressed (41). In contrast, in BCG-induced rodent models of TI, authors stressed the role of IFN-γ (42). Nevertheless, this concept can explain the lasting effects of MIA on rodent offspring.

In our previous study, we assessed the link between changes in mitochondrial respiration in PBMCs and innate immune responses in ASD. For screening of innate immune responses, we assessed PBMo cytokine profiles, since PBMo are major innate immune cells in the peripheral blood. Our results revealed the adaptive changes in mitochondrial respiration that we expected, in at least some ASD PBMCs (10). Moreover, we observed that such changes were associated with differences in IL-1ß/IL-10 ratios produced by PBMo and other PBMo cytokines profiles (10). Expression of miRNA in PBMo also differed in the IL-1ß/IL-10-based ASD subgroups (9). In our experience, ASD subjects whose PBMo revealed either high or low IL-1ß/IL-10 ratios tended to reveal repeatedly worsening behavioral symptoms following immune insults (typically microbial infection). Recent research in TI indicates that these findings may be explained by maladapted TI, following an initial immune challenge. Therefore, assessing changes in IL-1ß/IL-10 ratios may be helpful in addressing the role of innate immunity-induced chronic inflammation and possible maladapted TI in ASD.

Measurement of IL-1ß/IL-10 ratios produced by PBMo requires a fair amount of blood, and for this assay, immune cells in the blood samples need to be processed promptly. This makes it difficult to use the IL-1ß/IL-10 ratios by PBMo as a screening measure. Biomarkers found in the serum are better at fulfilling the requirements for screening measures. Namely, serum markers are easy to access, requiring a small amount of blood, and there is no need for quick processing; serum samples can be stored frozen for a prolonged time until measurement. ASD subjects identified by such serum biomarkers could then be further studied in more detail for innate immune abnormalities.

Recent research revealed that serum miRNAs serve as mediators of innate immunity, affecting functions of cells that have taken up exosomal miRNAs, with major sources of serum miRNAs being platelets and monocyte/macrophage lineage cells (13). Previously, we found altered miRNA expression by PBMo in the IL-1ß/IL-10 ratio–based ASD subgroups (9). It is also known that secreted miRNAs remain stable as a form of exosome, as opposed to serum cytokines, which have short half-lives in general. We thus reasoned that serum miRNA can serve as a surrogate biomarker, reflecting changes in innate immune responses in ASD. This study examined serum miRNA levels in ASD and non-ASD controls and compared these results with PBMo cytokine profiles and mitochondrial respiration by PBMCs. Mitochondrial respiration in PBMCs was assessed, since TI-induced metabolic changes in innate immune cells were expected to affect mitochondrial respiration, as detailed in the previous paragraph.

When ASD serum samples as a whole were compared to non-ASD controls, more than two-fold differences in expression were found in several miRNAs: 4 increased and 14 decreased. However, when miRNA levels were examined in the IL-1ß/IL-10 ratio–based ASD subgroups, only the high-ratio ASD subgroup revealed increase in expression of eight miRNAs, as compared to non-ASD controls. In the normal- and low-ratio ASD subgroups, the miRNAs that differed from non-ASD controls were all decreased in expression. The low-ratio ASD subgroup revealed a decrease of more miRNAs as compared to the normal-ratio ASD subgroup (18 *vs*. 10) (**Table 5**). Since the IL-1ß/IL-10 ratio–based ASD subgroups revealed changes in parameters of mitochondrial respiration consistent with our previous findings (**Supplementary Table 2**), our results indicated that changes in serum miRNA levels could reflect changes in IL-1ß/IL-10 ratios in PBMo and mitochondrial respiration in PBMCs.

To further address a link between serum miRNA levels and changes in innate immune responses, we assessed whether genes targeted by miRNAs whose serum levels were altered in the IL-1ß/IL-10-based ASD subgroups are enriched in specific signaling pathways. miRNA targeted genes were enriched in pathways of cell proliferation and cell differentiation in all the IL-1ß/IL-10 ratio–based ASD subgroups. In addition, targeted genes are enriched in signaling pathways of neuronal development and synaptic plasticity (neurotrophin signaling and axon guidance pathways) in all the ASD subgroups. Therefore, part of serum miRNA expression in ASD may reflect altered neuronal development and synaptic plasticity in ASD, as proposed in the pathogenesis of neuropsychiatric conditions including ASD (44).

When targeted genes were analyzed in the IL-1ß/IL-10 ratio–based ASD subgroups, in the high- and low-ratio ASD subgroups, targeted genes were enriched in signaling pathways of key monocyte functions such as cell adhesion, migration, secretion, and immune cell activation/regulation (mTOR pathway, and TGF-ß signaling pathways). Furthermore, in the high IL-1ß/IL-10 ratio ASD subgroup, targeted genes were also enriched in pathways of an insulin signaling pathway; this pathway is likely associated with the reprogramming process in TI. The pathways critical for monocyte functions were enriched by genes targeted by miRNAs altered in the high and low IL-1ß/IL-10 ratio ASD subgroups, and these pathways are also important for brain function, as has been best described for the mTOR pathway (45). Neuronal development is intricately affected by inflammation (46, 47) and altered innate immune responses have been reported in other neurodegenerative diseases (48–50). In summary, our results indicate that changes in serum miRNA levels in ASD could reflect changes in monocyte functions associated with TI and subsequently affect mitochondrial functions in the high and low IL-1ß/IL-10 ratio ASD subgroups.

We then assessed if there was any linear correlation(s) between IL-1ß/IL-10 ratios and mitochondrial respiration, with serum miRNA levels. Consistent with the results of target gene analysis, we found significant differences in these correlations across the IL-1ß/Il-10-based ASD subgroups as compared to non-ASD controls. For example, correlations between IL-1ß/IL-10 ratios and miRNA levels are much more evident in non-ASD controls (**Table 8**). As for correlations between mitochondrial respiration and serum miRNA levels, we also found close correlations between mitochondrial parameters and several miRNAs (negative and positive associations) in non-ASD controls (**Table 12**). On the other hand, such correlations were less evident in the ASD subgroups (**Table 12**). We previously observed that ASD PBMCs revealed maladaptive changes in mitochondrial respiration (10), so that changes in serum miRNA levels, especially in the low IL-1ß/IL-10 subgroup, can be associated with such maladaptive changes.

We also evaluated correlations between each of the PBMo cytokines produced and the serum miRNA levels in the ASD subgroups and non-ASD controls. Distinct differences were observed across the IL-1ß/IL-10-based ASD subgroups and were most evident in TNF-α, IL-10, and CCL2 production. Specifically, TNF-α production was positively and negatively correlated with multiple serum miRNA levels in non-ASD controls (**Table 10**), but correlations between these two parameters were less apparent in the ASD subgroups. There were predominant negative correlations between IL-10 levels and serum miRNAs in the non-ASD controls, while much less correlations were noted in the high- and low-ratio ASD subgroups (**Table 9**). In the non-ASD controls, CCL2 production was negatively and positively correlated with serum levels of several miRNAs, while in the low IL-1ß/IL-10 ratio ASD subgroup, mostly positive correlations were observed (**Table 11**). Less of an correlation was also observed between CCL2 production and serum miRNA levels in the normal- and high-ratio ASD subgroups (**Table 11**). CCL2 is a major chemokine produced by monocytes, and changes in plasma levels of CCL2 have been reported in ASD (51, 52). It is of note that in non-ASD controls, miRNAs that revealed correlations with monocyte cytokine parameters revealed correlations in multiple culture conditions.

Taken together, our results indicate that IL-1ß/IL-10 ratio–based ASD subgroup-specific associations exist between levels of several serum miRNAs and PBMo cytokine profiles. Combining the findings with target gene analysis, our results indicate that certain serum miRNA levels may reflect reprogramming or TI of innate immunity in ASD, most likely in the high and low IL-1ß/IL-10 ratio ASD subgroups. miRNAs that revealed marked differences in associations with IL-1ß/IL-10 ratios, production of TNF-α, IL-10, and CCL2 by PBMo across the ASD subgroups were detected in relatively high amounts in the serum (miR134-5p, miR-382-5p, miR-103a-5p, miR-378-3p, mi-423-5p, miR100-5p, and miR 99b-5p) (**Table 2**). The first five miRNAs also revealed differences among the ASD subgroups (**Table 6**), and these results were not affected by clinical variables. These miRNAs may be candidate biomarkers for assessing innate immune abnormalities associated with TI in ASD subjects.

As summarized in the demographic information of the IL-1ß/IL-10 ratio–based ASD subgroups (**Table 4**), ASD subjects in the low IL-1ß/IL-10 ratio subgroup had a higher frequency of non-IgE-mediated FA, than normal ASD groups, consistent with our previous results (9, 10). We also observed that ASD subjects with low IL-1ß/IL-10 ratios are often sicker and vulnerable to multiple, unrelated immune insults. In these subjects, maladapted TI may play an important role. Future prospective and longitudinal studies involving a larger number of ASD subjects will provide interesting and important information on how serum miRNA levels are associated with changes in innate immune responses (possibly TI). Findings of such studies may lead to the application of currently available immunomodulating agents for the treatment of certain ASD patients.

## Data Availability

Clinical features of the ASD are available through NDAR data base (https://ndar.nih.gov/). The additional datasets generated for this study are available on request to the corresponding author.

## Ethics Statement

The study followed the protocols approved by the Institutional Review Board, Saint Peter’s University Hospital, New Brunswick, NJ, United States. In this study, both ASD and non-ASD TD subjects were enrolled, and the signed consent forms were obtained prior to entering the study. Consent was obtained from parents if participant was a minor (<18 years old) or parents had custody. 

Although this study involves children and young adults diagnosed with ASD, the involved procedures *per se* were considered to be minimally hazardous, since this study only involved venipuncture, chart review, and request of filling out questionnaires by parents.

## Author Contributions

HJ was responsible for the study design, recruitment of the study subjects, collection of clinical information and blood samples, analysis of the overall data, and preparation of most of this manuscript. LG conducted cytokine production assays with the use of purified monocytes and also prepared samples for mitochondrial function for shipping to RF’s laboratory and miRNA extraction from serum samples. GT conducted gene target analysis and contributed manuscript preparation from the view of molecular geneticist. SR and SB conducted assays for mitochondrial respiration with the use of PBMCs and helped prepare a manuscript. RF supervised SR and SB and discussed with HJ extensively, regarding data analysis and helped manuscript preparation.

## Funding

This study was supported by funding from Autism Research Institute, San Diego, CA, Jonty Foundation, St. Paul, MN, and the Governor’s Council for Medical Research and Treatment of Autism, DHHS, Trenton, NJ.

## Conflict of Interest Statement

SR and RF are listed as collaborators in the BioROSA Technologies, Inc.

The remaining authors declare that the research was conducted in the absence of any commercial or financial relationships that could be construed as a potential conflict of interest.

## Abbreviations

Ab, antibody; ABC, aberrant behavior checklist; AC, allergic conjunctivitis; ADHD, attention deficiency hyperactivity disorder; ADI-R, autism diagnostic inventory, revisited; ADOS, autism diagnostic observational scale; AED, anti-epileptic drugs; Ag, antigen; ALR, ATP-linked respiration; ANOVA, analysis of variance; AR, allergic rhinitis; ASD, autism spectrum disorder; CCL2, CC-chemokine-ligand-2; CPM, count per minute; CSHQ, Children’s sleep habit questionnaire; DE, differential expression; ELISA, enzyme linked immune-sorbent assay; ETC, electron transport chain; FA, food allergy; FDR, false discovery rate; IL, interleukin; LPS, lipopolysaccharide; MIA, maternal immune activation; miRNA, microRNA; MRC, maximum respiration capacity; mTOR, mammalian target of rapamycin; NFA, non-IgE-mediated food allergy; OCR, oxygen consumption rate; PBMCs, peripheral blood mononuclear cells; PBMo, peripheral blood monocytes; PLR, proton-linked respiration; PST, prick skin testing; RC, reserve capacity; SAD, specific antibody deficiency; SD, standard deviation; SSRI, selective serotonin receptor inhibitor; SPUH, Saint Peter’s University Hospital; TGF, transforming growth factor; TLR, toll-like receptor; TMM, trimmed mean of M values; TNF, tumor necrosis factor; Treg cells, regulatory T cells; VABS, Vineland adaptive behavioral scale.
