# Supplementary material for: Serum microRNAs in ASD: Association With Monocyte Cytokine Profiles and Mitochondrial Respiration
Source: Front Psychiatry. 2019 Sep 10;10:614. doi: 10.3389/fpsyt.2019.00614 (PMC6748029; doi:10.3389/fpsyt.2019.00614)
Supplement: Supplementary file 1 [file Table_1.pdf]

Table 1 Effects of Gender and medication use on serum miRNA levels

Clinical features	ASD subjects		Mann-Whitney
Gender	Male (98)	Female (18)	p-value
miR-4732-5p	86.1±15.5	139.1±12.5	0.0295
miR-423-3p	56272±61862	40093±52025	0.035
Neuroleptics	No (N=104)	Yes (N=12)	p-value
miR-233-5p[2]	88.2±87.3[3]	140.3±74.5	0.0033
miR-3614-5p[2]	34.2±59.7	57.3±56.3	0.01441
miR-370-3p	183.2±254.0	85.7±65.7	0.0292
miR 99b-5p	9328.3±6842.5	5931.3±1727.0	0.0467
ADHD[1] meds	No (N=102)	Yes (N=14)	p-value
miR-7-5p[2]	148.4±179.9	81.7±58.4	0.0424
miR-103a-3p[2]	644.1±327.5	484.3±207.4	0.0399
AEDs	No (N=86)	Yes (N=30)	p-value
miR 206[2]	429.7±3462.7	54.8±50.3	0.0024
miR 320b	666.4±713.0	451.4±232.1	0.0414
SSRIs	No (N=97)	Yes (N=19)	p-value
miR 576-3p[2]	103.2±201.6	26.2±30.3	0.0236
miR 433-3p	15.5±12.7	27.6±19.9	0.00439
miR-20a-5p	310.9±570.7	192.9±125.2	0.02571

[1] Abbreviations used: ADHD, attention deficiency hyperactivity disorder; AEDs, anti-epileptic drugs; SSRIs, selective serotonin receptor inhibitors.
[2] miR-233-5p, miR-3614-5p, miR-7-5p, miR-103a-3p, miR 206, miR-576-3p were differentially expressed in the serum as compared to non-ASD controls.
[3] Serum miRNA levels were expressed as TMM normalized read counts ± SD as detailed in the methods section.

Suppl. Table 1. Differences of miRNA levels due to gender, and the use of medications (neuroleptics, ADHD medications, AEDs, and SSRIs)
