# Supplementary material for: Serum microRNAs in ASD: Association With Monocyte Cytokine Profiles and Mitochondrial Respiration
Source: Front Psychiatry. 2019 Sep 10;10:614. doi: 10.3389/fpsyt.2019.00614 (PMC6748029; doi:10.3389/fpsyt.2019.00614)
Supplement: Supplementary file 2 [file Table_2.pdf]

Table 2 IL-1ß/IL-10 ratios produced by PBMo under various culture conditions and mitochondrial respiration parameters by PBMCs in ASD subgroups and non-ASD controls in this study.

	IL-1ß/IL-10 ratio based ASD subgroups High (N=47)	Normal (N=43)	Low (N=25)	Non-ASD Control (N=34)	Statistics[1]
IL-1ß/IL-10 ratios					
medium	1.53±0.89	0.86±0.62	0.32±0.19	0.86±0.62	<0.00001
LPS	3.28±1.20	1.15±1.10	0.79±0.59	2.1±1.02	<0.00001
zymosan	6.17±4.88	2.62 ±2.52	1.73±1.78	3.61±2.62	<0.00001
CL097	10.86±5.55	3.73±2.50	3.02±2.97	5.08±4.26	0.00012
Mitochondrial Respiration in PBMCs					
ALR/PLR	5.87±8.21	8.19±15.13	5.07±5.14	3.53±5.64	0.5886
MRC/PLR	22.43±31.82	29.25±49.91	17.07±21.33	9.45±14.09	0.0438
MRC	93.50±57.72	112.24±64.93	85.77±59.26	68.55±37.76	0.00411
RC	60.72±50.17	73.08±52.33	51.30±50.04	33.59±30.84	0.00633

[1]Krushkal Wallis test was used for assessing differences. Results were expressed as a mean value ± SD

Suppl. Table 2: Results of IL-1ß/IL-10 ratios produced by PBMo under various culture conditions and mitochondrial respiration by PBMCs in the IL-1ß/IL-10 based ASD subgroups, and non-ASD controls. As for mitochondrial respiration parameters, MRC/PLR, MRC, and RC were found to differ among the above described study groups by Krushkal Wallis test, consistent with our previous report.
